# High-Throughput COVID-19 Testing of Naso-Oropharyngeal Swabs Using a Sensitive Extraction-Free Sample Preparation Method

**DOI:** 10.1128/spectrum.01358-22

**Published:** 2022-08-11

**Authors:** Todd M. Pryce, Erin J. Haygarth, Jessica Bordessa, Courtney T. Jeffery, Ian D. Kay, James P. Flexman, Peter A. Boan

**Affiliations:** a PathWest Laboratory Medicine WA, Department of Clinical Microbiology, Fiona Stanley Hospital, Murdoch, Western Australia, Australia; b Department of Infectious Diseases, Fiona Stanley Fremantle Hospital Group, Murdoch, Western Australia, Australia; University of Utah and ARUP Laboratories

**Keywords:** PlexPCR, cobas, SARS-CoV-2, extraction-free, high-throughput

## Abstract

High-throughput diagnostic assays are required for large-scale population testing for severe acute respiratory coronavirus 2 (SARS-CoV-2). The gold standard technique for SARS-CoV-2 detection in nasopharyngeal swab specimens is nucleic acid extraction followed by real-time reverse transcription-PCR. Two high-throughput commercial extraction and detection systems are used routinely in our laboratory: the Roche cobas SARS-CoV-2 assay (cobas) and the Roche MagNA Pure 96 system combined with the SpeeDx PlexPCR SARS-CoV-2 assay (Plex). As an alternative to more costly instrumentation, or tedious sample pooling to increase throughput, we developed a high-throughput extraction-free sample preparation method for naso-oropharyngeal swabs using the PlexPCR SARS-CoV-2 assay (Direct). A collection of SARS-CoV-2-positive (*n* = 185) and -negative (*n* = 354) naso-oropharyngeal swabs in transport medium were tested in parallel to compare Plex to Direct. The overall agreement comparing the qualitative outcomes was 99.3%. The mean cycle of quantification (*C_q_*) increase and corresponding mean reduction in viral load for Direct ORF1ab and RdRp compared to Plex was 3.11 *C_q_* (−0.91 log_10_ IU/mL) and 4.78 *C_q_* (−1.35 log_10_ IU/mL), respectively. We also compared Direct to a four-sample pool by combining each positive sample (*n* = 185) with three SARS-CoV-2-negative samples extracted with MagNA Pure 96 and tested with the PlexPCR SARS-CoV-2 assay (Pool). Although less sensitive than Plex or Pool, the Direct method is a sufficiently sensitive and viable approach to increase our throughput by 12,032 results per day. Combining cobas, Plex, and Direct, an overall throughput of 19,364 results can be achieved in a 24-h period.

**IMPORTANCE** Laboratories have experienced extraordinary demand globally for reagents, consumables, and instrumentation, while facing unprecedented testing demand needed for the diagnosis of SARS-CoV-2 infection. A major bottleneck in testing throughput is the purification of viral RNA. Extraction-based methods provide the greatest yield and purity of RNA for downstream PCR. However, these techniques are expensive, time-consuming, and depend on commercial availability of consumables. Extraction-free methods offer an accessible and cost-effective alternative for sample preparation. However, extraction-free methods often lack sensitivity compared to extraction-based methods. We describe a sensitive extraction-free protocol based on a simple purification step using a chelating resin, combined with proteinase K and thermal treatment. We compare the sensitivity qualitatively and quantitatively to a well-known commercial extraction-based system, using a PCR assay calibrated to the 1st WHO international standard for SARS-CoV-2 RNA. This method entails high throughput and is suitable for all laboratories, particularly in jurisdictions where access to instrumentation and reagents is problematic.

## INTRODUCTION

Diagnostic tools are essential to manage the current coronavirus disease 2019 (COVID-19) pandemic, and reliable, high-throughput laboratory tests are required (1). These tools are the strategic cornerstone to mitigate severe acute respiratory syndrome coronavirus 2 (SARS-CoV-2) spread, facilitating early diagnosis, isolation of infected individuals, and clearance of essential personnel to continue to work (2). Since 20 March 2020, we have performed more than 700,000 tests using two commercial extraction and detection systems: the cobas SARS-CoV-2 assay (Roche Molecular Systems, Branchburg, NJ, USA), and the PlexPCR SARS-CoV-2 assay (SpeeDx, Eveleigh, NSW, Australia) (3–6). The PlexPCR SARS-CoV-2 assay workflow in our laboratory utilizes a maximum of four MagNA Pure 96 instruments (Roche) for RNA extraction, two PlexPrep liquid handlers (SpeeDx) for 384-well PCR plate preparation, and four LightCycler 480 thermal cyclers (Roche) for amplification and detection. Using both commercial systems, we performed 4,324 tests over a 30-h period (24-h hands-on) in a recent surge testing event, with the PlexPCR SARS-CoV-2 workflow demonstrating 155% higher throughput than cobas SARS-CoV-2 (5). That surge testing event and others since have shown that we have additional PlexPrep and thermal cycling capacity; our testing throughput is limited by sample handling and the capacity of the extraction-based systems. Our 24-h extraction-based testing capacity is estimated to be 1,316 results for cobas SARS-CoV-2 (14 runs with 94 samples per run) and 6,016 results for PlexPCR SARS-CoV-2 (64 MagNA Pure 96 runs with 94 samples per run for 16 PlexPCR runs with 376 samples per run). If fully utilized, the additional thermal cycler capacity could add more than 12,000 results in a 24-h period. Sample pooling is considered a viable strategy for increased testing capacity while in a low-prevalence setting (7). However, with the inevitability of testing surges and with testing capacity gains from pooling diminishing at high disease prevalence, alternative strategies were investigated. These included additional instrumentation—requiring significant expenditure and laboratory space—or rapid extraction-free methods of sample preparation. Extraction-free methods result in a loss in sensitivity compared to extraction-based methods (8–12); however, pooled testing strategies also demonstrate loss in sensitivity compared to single-specimen testing, depending on the sample pool size (7, 13). The key differences between single-specimen testing methods compared to pooled-specimen approaches are that single-specimen testing methods are not affected by high disease prevalence, do not require laborious positive-pool retesting, and allow test turnaround time to be maintained (7). After considering our options, we sought to increase the single-specimen testing capacity by developing a rapid extraction-free sample preparation method for the PlexPCR SARS-CoV-2 workflow. A pre-implementation study was performed using a panel of stored positive and negative samples. We assessed the relative sensitivity of the optimized extraction-free method and compared it to that of the MagNA Pure 96 extraction method for undiluted samples and a four-sample pool by using the PlexPCR SARS-CoV-2 assay. To standardize cycle of quantification (*C_q_*) analysis, we developed an in-house quantitative method using standards prepared from the 1st WHO international standard (IS) for SARS-CoV-2 RNA. Further analysis was performed to assess the mean change in the *C_q_* or reduction of viral load of the extraction-free method compared to that with the MagNA Pure 96 extraction method.

## RESULTS

All raw results (*C_q_* values), including the calculated quantitative results and the SARS-CoV-2 IS curves, are presented in the supplemental material.

### Quantitative standards.

The *C_q_* values for each SARS-CoV-2 IS concentration and Plex ORF1ab and RdRp standard curves are provided in the supplemental material. ORF1ab demonstrated an *r*^2^ value of 1.0 over the range of 2.70 to 6.70 log_10_ IU/mL. ORF1ab detection at 0.70 and 1.70 log_10_ IU/mL was not reproducible and was omitted from the standard curve. RdRp demonstrated an *r*^2^ value of 0.998 over the range of 2.70 to 6.70 log_10_ IU/mL. RdRp detection at 0.70 and 1.70 log_10_ IU/mL was not reproducible and was omitted from the standard curve. The Plex PCR targets standard curves demonstrated a high degree of linearity and were commutable within 0.23 log_10_ IU/mL over the range tested.

### Qualitative comparison.

The qualitative results comparing the Direct and Plex methods are summarized in Table 1. The overall agreement comparing Direct to Plex was 99.3% (535/539; confidence interval [CI], 98.1% to 99.7%). As shown in the supplemental material, four samples were negative with Direct (samples 72, 76, 106, and 147). Corresponding Plex *C_q_* values ranged between 20.77 and 23.12 *C_q_* for ORF1ab (<3.77 log_10_ IU/mL) and between 20.01 and 23.25 *C_q_* for RdRp (<3.60 log_10_ IU/mL). Four samples were positive for a single target when tested with Direct. Three were RdRP positive only (samples 65, 78, and 145) and one was ORF1ab positive only (sample 120). The qualitative results for the positive samples comparing Pool to Plex demonstrated 100% concordance. However, two samples (72 and 78) were positive for a single target (ORF1ab) when tested with Pool.

**TABLE 1:** Results obtained with the Direct and Pool methods in comparison to results with Plex^*a*^

Method	Detected	Not detected	% PPA (95% CI)	% PNA (95% CI)	% POA (95% CI)	Total
Direct	181	358	97.8 (94.6–99.2)	100 (98.9–100)	99.3 (98.1–99.7)	539
Pool	185	354	100 (98.0–100)	100 (98.9–100)	100 (99.3–100)	539

a Detected and not detected columns report the number of positive and negative results, respectively. PPA, percent positive agreement; PNA, percent negative agreement; POA, percent overall agreement.

### Quantitative comparison.

The mean change in *C_q_* and log_10_ IU/mL for Pool and Direct compared to Plex are shown in Table 2. The mean *C_q_* and log_10_ IU/mL change for Pool ORF1ab and RrRp compared to Plex was 1.64 *C_q_* (−0.48 log_10_ IU/mL) and 1.74 *C_q_* (−0.49 log_10_ IU/mL), respectively. The mean log_10_ IU/mL change was quantitatively very similar for each target, differing by 0.10 log_10_ IU/mL. Pool ORF1ab detected two more samples than Pool RdRp (185 compared to 183). The mean *C_q_* and log_10_ IU/mL change for Direct ORF1ab and RdRp compared to Plex was 3.11 *C_q_* (−0.91 log_10_ IU/mL) and 4.78 *C_q_* (−1.35 log_10_ IU/mL), respectively. A larger difference, 0.44 log_10_ IU/mL, was observed for each target, with Direct RdRp showing the greatest change in *C_q_*. However, despite the greater change in *C_q_*, Direct RdRp qualitatively detected more samples than Direct ORF1ab (180 compared to 178).

**TABLE 2:** Mean change in *C_q_* and reduction in viral load detected with the Pool and Direct methods compared to results with Plex^*a*^

Method	PlexPCR target	No. compared	Mean Δ*C_q_* (95% CI)	Mean Δlog_10_ IU/mL (95% CI)	*P* value
Pool	ORF1ab	185	1.64 (1.42 to 1.87)	−0.48 (−0.55 to −0.42)	<0.05
Pool	RdRp	183	1.74 (1.56 to 1.92)	−0.49 (−0.54 to −0.44)	<0.05
Direct	ORF1ab	178	3.11 (2.83 to 3.39)	−0.91 (−0.99 to −0.83)	<0.05
Direct	RdRp	180	4.78 (4.52 to 5.04)	−1.35 (−1.42 to −1.27)	<0.05

a Summary data and two-tailed paired *t* test results (*P* < 0.05), showing the mean change in *C_q_* or log_10_ IU/mL for the Pool and Direct methods, compared to Plex.

## DISCUSSION

Real-time reverse transcription-PCR combined with purified RNA from samples is the gold standard method for SARS-CoV-2 detection. The development of extraction-free methods during the early days of the COVID-19 pandemic was largely driven by the lack of reagents for RNA extraction, with the added benefit of reduced cost and speed. In contrast, we developed the Direct method to improve throughput as our primary goal. With the Western Australia borders closed for nearly 2 years, sufficient time has elapsed for the reagent and consumable supply chains to be restored. We sought to utilize additional testing capacity of the liquid handlers and the thermal cyclers while overcoming the RNA extraction bottleneck. If testing demand increased above our extraction-based capacity, we could divert samples to the Direct method if required.

Extraction-free methods for use with nasopharyngeal specimens have been investigated in a number of pilot studies (8, 10, 11, 14–16). The overall sensitivity comparing extraction-free methods to extraction-based methods vary from 55 to 99% according to the PCR assays evaluated. These studies are limited by the low number of samples tested (both positive and negative) and reported different sensitivities when stratifying *C_q_* values obtained with the reference method. Investigations with a larger number of samples have reported sensitivities of 96% (*n* = 597, no *C_q_* stratification) (9) and 95% (*n* = 155 positive samples with *C_q_* values of <33) (17). These investigations used heat as a sample pretreatment step for virus inactivation and operator safety (95°C for 5 to 10 min), followed by the addition of lysate directly into the PCR mixture. Optimization experiments have shown that high temperature and short durations (95 to 98°C for 5 to 15 min) improve (reduce) *C_q_* values compared to lower temperature and longer durations (60°C for 30 min) (9). Heat inactivation prior to testing with extraction-based methods also reduces the sensitivity of SARS-CoV-2 RNA detection (4, 18, 19). Other investigators have shown the addition of proteinase K (55°C for 15 min) followed by heat treatment also improves *C_q_* values up to three cycles compared to heat treatment alone (11). Based on the correlation between heat and reduced yield, a method designed to minimize the total time of SARS-CoV-2 exposure to elevated temperatures across the entire procedure would be advantageous. The extraction-free method we developed incorporates an optimal proteinase K concentration, a proteinase K incubation step at the lowest temperature that does not compromise yield, and a heat step at the highest temperature and shortest duration possible for proteinase K inactivation. We also included an optimized concentration (8 to 9% final concentration) of Chelex-100 ion exchange resin in a low concentration of Tris-HCl buffer (10 mM Tris-HCl), which has been shown to dramatically improve sensitivity (12). The inclusion of Chelex-100 resin is particularly important, since the chemical and biological constituents of the viral transport media (such as salts and denatured proteins) are directly transferred into the PCR mix; Chelex-100 removes free ions and positively charged contaminants in solution that may affect polymerase activity or specificity and preserves SARS-CoV-2 RNA in the sample by binding cofactors required for nucleases (12). As extraction-free approaches are competing with extraction-based methods in terms of purity and yield, the final goal is to achieve the highest RNA yield possible compared to the reference method. During assay optimization, RNA yield was measured with quantitative PCR. Hence, we primarily focused on sensitivity and yield during assay development, avoiding extended exposure to heat; SARS-CoV-2 exposure for laboratory personnel can be mitigated with adherence to strict laboratory procedures and personal protective equipment. Combining these elements, we benchmarked a method which included proteinase K at a final concentration of 0.58 mg/mL, an incubation step of 37°C for 10 min, and then 95°C for 90 s for heat inactivation of proteinase K. The duration of 90 s was the minimum time required to completely inactivate the proteinase K concentration used (which is essential).

Following optimization of the Direct method, we benchmarked the method against the routine Plex method, which uses MagNA Pure for extraction. As expected, we observed a loss in sensitivity of the Direct method compared to Plex both qualitatively and quantitatively. Despite the loss in analytical sensitivity, the overall agreement was 99.3% combined without *C_q_* stratification of results. We note that other studies have focused on positive sample comparisons and the number of negative samples tested with extraction-free approaches has been limited or absent (8, 9, 11, 18). Diagnostic PCR assays are optimized for purified nucleic acids in terms of primer-probe stringency and test performance. Compared to extraction-based methods, crude lysates may contain more interfering substances, including salts carried over from the transport media. PCR assays need to be thoroughly tested for nonspecific primer-probe interactions when modifying the method of template preparation. We included a large number of positive and negative samples in this evaluation. We did not observe any nonspecificity or PCR inhibition for any of the samples tested. Furthermore, given the high positive prevalence of the population tested, the extraction-free method must exhibit a high degree of specificity and not rely on extraction-based approaches for SARS-CoV-2 confirmation; the extraction-free method must be robust, high-throughput and stand alone as a single diagnostic test. During this study and the subsequent training of staff, we found the Direct method to be robust and reliable.

To further investigate the loss in sensitivity for the Direct method compared to Plex and Pool, we performed quantitative analysis of the *C_q_* values using the 1st WHO international standard for SARS-CoV-2 RNA. The mean reduction in viral load for Direct ORF1ab and RdRp compared to Plex was −0.91 and −1.35 log_10_ IU/mL, respectively. Compared to a mathematical model, a sample with a SARS-CoV-2 concentration of 5.00 log_10_ IU/mL extracted with MagNA Pure (200 μL), eluted in 50 μL, with 2.5 μL then used as template for PCR, would contain 3.00 log_10_ IU per reaction mixture (assuming 100% efficiency of extraction). The same sample tested with Direct (100 μL), added to 40 μL of the 30% Chelex-100–proteinase K–internal control mixture, with 2.5 μL then used as template for PCR, would contain 2.25 log_10_ IU per reaction mixture (assuming 100% efficiency of lysis). Therefore, a loss of 0.75 log_10_ IU per reaction mixture (25%) based on template volume would be expected. Nonetheless, the Direct method performed well qualitatively against 185 positive samples, with a mean viral load of 6.00 log_10_ IU/mL when tested with Plex. Eleven Plex samples contained <3.76 log_10_ IU/mL (≈20.8 *C_q_*), and the Direct method failed to detect four samples below this value. The PlexPCR 20.8 *C_q_* value was approximately equivalent to a cobas *C_q_* of 31.5, based on previous work (5). We acknowledge that caution should be taken when comparing *C_q_* values with different assays for quantifying SARS-CoV-2 RNA (20). However, SARS-CoV-2 is unlikely to be recovered from culture at late cycle threshold values, which carries less importance for viral transmission (21). The loss of sensitivity at this level may be an acceptable trade-off for higher throughput. Where sensitivity is a primary clinical concern (when screening solid organ donors and candidates or on an immunocompromised host ward), one may elect to continue with extraction-based methods.

In conclusion, the Direct method resolves throughput bottlenecks and adds an additional 12,032 results per day, comprising 128 Direct plates of 94 samples per plate, for an additional 32 PlexPCR runs with 376 samples per run. This complements our existing maximum of 7,332 per day with the extraction-based methods, to achieve a total of 19,364 results in a 24-h period. This method is useful to the wider scientific community as an alternative to RNA extraction, particularly for jurisdictions with issues with reagent supply or a lack of instrumentation (22). Finally, while less sensitive than the gold standard extraction-based method, this extraction-free method represents a viable, high-throughput diagnostic approach, with a degree of sensitivity that is suitable for COVID-19 testing.

## MATERIALS AND METHODS

### Study setting.

The Department of Clinical Microbiology Laboratory (PathWest, Fiona Stanley Hospital, Murdoch) is a reference laboratory located in Perth, Western Australia. During the early stages of the pandemic, samples were tested using the cobas SARS-CoV-2 assay (Roche catalog number 09175431190) as the primary testing method. Thermal pretreatment of the sample was performed before cobas testing (3, 4). We implemented the PlexPCR SARS-CoV-2 assay (SpeeDx catalog number 1301384) in September 2020 as an additional testing method to increase testing capacity (5). All samples were naso-oropharyngeal swabs collected according to a combined nasopharyngeal and oropharyngeal swab procedure (23). Swabs were placed in Copan UTM-RT medium (Copan, Brescia, Italy), CITOSWAB (Citotest Scientific Jiangsu, People’s Republic of China), or PathWest virus transport medium (VTM) (24). In anticipation of increasing demand for testing, a head-to-head evaluation was performed comparing the MagNA Pure 96 extraction-based method (Plex) with the optimized extraction-free method (Direct). We also compared the results to a four-sample pool also tested with PlexPCR using the MagNA Pure 96 method (Pool). The testing was performed on 8 February 2022 and 9 February 2022. All samples were tested in parallel.

### Samples tested.

Positive samples were collected from 30 January 2021 to 8 February 2022. All SARS-CoV-2-positive samples were stored at −80°C as aliquots from the remaining original sample. Negative samples were collected 3 February 2022. Due to −80°C storage constraints, all negative samples were stored at 4°C in the original transport media tube. Samples positive for cobas ORF1ab and E-gene were defined as SARS-CoV-2 detected. Similarly, samples positive for Plex ORF1ab and RdRp were defined as SARS-CoV-2 detected. Samples positive for a single target were reflexively tested using Xpert Xpress SARS-CoV-2 (Xpert; Cepheid, Sunnyvale, CA, USA) from the original sample (not thermally treated). Samples positive for at least one different target compared to cobas or Plex were defined as SARS-CoV-2 detected. All other results including negative Xpert results were considered equivocal for SARS-CoV-2, and repeat collections were performed. For the preimplementation study, we selected 185 consecutive positive samples and 354 consecutive negative samples. No equivocal SARS-CoV-2 samples were used in the preimplementation study, as these were not considered true positives for the purposes of method comparison.

### Plex method.

All positive and negative samples tested with Plex were extracted using MagNA Pure 96 DNA and viral nucleic acids small-volume kit (Roche catalog number 06543588001) using the Pathogens Universal 200 protocol (version 4.0). Twenty microliters of the PlexPCR internal control (SpeeDx catalog number 1301384) was added to each sample using the MagNA Pure 96 internal control tube (Roche catalog number 06374905001). A sample input volume of 200 μL and an elution volume of 50 μL were used. PlexPrep processing, including PlexPCR amplification and detection and result analysis, was performed following the protocols issued by the manufacturer.

### Four-sample pool preparation method.

For the Pool method, 50 μL of each positive sample (*n* = 185) was combined with three separate 50-μL aliquots from different SARS-CoV-2-negative patient samples (*n* = 555 samples in total) previously tested with cobas. All samples in the four-sample pool method were extracted and tested as described above for Plex.

### Direct method.

We optimized the Direct method prior to the preimplementation study in terms of analytical sensitivity, efficient reagent use, and throughput. A sensitive extraction-free method using a chelating resin was optimized for sample preparation based on the work of others and our own experience (12, 25, 26). A 10 mM Tris-HCl solution was prepared from a 1 M Tris-HCl solution (catalog number T2663; Sigma-Aldrich, Saint Louis, MO, USA) with molecular biology-grade water (catalog number W4502; Sigma-Aldrich). A 30% (wt/wt) suspension of Chelex-100 resin (catalog number 142-1253; Bio-Rad, Hercules, CA, USA) was prepared with 10 mM Tris-HCl. For 94 samples, 4.8 mL of the Chelex suspension was transferred to a secondary tube (Greiner Bio-One; catalog number 459000; Kremsmünster, Austria). To this suspension, 144 μL of a 20-mg/mL proteinase K solution (catalog number A5051; Promega, Madison, WI, USA) and 100 μL of PlexPCR internal control RNA (catalog number 1301384; SpeeDx) was added and mixed. Forty microliters of the Chelex reagent was added to each well of an Axygen PCR microplate (PCR-96-AB-C; Corning, New York, NY, USA) using a multichannel pipette and 200-gauge wide-bore pipette tips (2069G; Molecular BioProducts, San Diego, CA, USA). A 100-μL aliquot of each patient sample or control was added to each well without mixing. The Optitrol NAT SARS-CoV-2 reference material (NT04032; DiaMex, Heidelberg, Germany) was used as a positive control and VTM was used as a negative control. The plate was sealed with ThermalSeal film (100THERPLT; Excel Scientific, Victorville, CA, USA) and placed into a GeneAmp PCR system 9700 thermocycler (Applied Biosystems, Waltham, MA, USA) in a pre-PCR area. The incubation program consisted of 37°C for 10 min, 95°C for 90 s, and 4°C for 1 min. The lid was preheated to 103°C. Following the incubation program, the plate was removed and sealed in a zip-lock bag, transferred to a sealed centrifuge plate holder, and centrifuged at 4,000 rpm for 5 min using a Sigma 4-15 centrifuge (Sigma-Aldrich). The sealed centrifuge plate holders were opened in a class 2 biological safety cabinet, and the ThermalSeal film was removed. Processed plates (up to 4 at a time) were loaded into the PlexPrep for 384-well PCR plate preparation using appropriate personal protective equipment.

### Preimplementation study.

All PlexPrep runs were prepared using PlexPCR master mix of a single lot number. The master mix consisted of 5 μL Plex master mix (2×), 0.1 μL reverse transcriptase (100×), 0.2 μL RNase inhibitor (50×), 0.5 μL CoV-2 mix, and 1.7 μL nuclease-free water for a total of 7.5 μL per reaction mixture. The PlexPrep liquid handler was utilized for master mix dispensing (7.5 μL) to the LightCycler 480 384-well reaction plate. The nucleic acid extracts for Plex and Pool, including the lysates for Direct, were all added to each well (2.5 μL), also using the PlexPrep. PlexPCR amplification and detection and result analysis were performed following the protocols issued by the manufacturer. The PlexPCR amplification protocol includes a 10-cycle touchdown. No fluorescent acquisitions are performed during the touchdown cycles; hence, *C_q_* values are reported approximately 10 cycles earlier than with conventional real-time PCR (5). The *C_q_* values were recorded for ORF1ab, RdRp, and the internal control.

### Quantitative standards, external control, and analysis.

Quantitative standards were prepared from the 1st WHO international standard for SARS-CoV-2 (NISBSC code 20/146), supplied as 7.70 log_10_ IU/mL (National Institute for Biological Standards and Control, Hertfordshire, UK). The standard was reconstituted with 0.5 mL of phosphate-buffered saline following the manufacturer’s instructions. Once reconstituted, the standard was 10-fold serially diluted in a naso-oropharyngeal matrix. This matrix consisted of pooled naso-oropharyngeal samples from samples that previously tested negative for SARS-CoV-2 using cobas. Seven standards were prepared over the range of 0.70 to 6.70 log_10_ IU/mL. Each standard was tested in triplicate with Plex using the same amplification and detection lot number used for patient samples. The mean *C_q_* value at each concentration was used to calculate ORF1ab and RdRp standard curves and regression. At least two positive replicates at each dilution were required to be included in the standard curve. The regression formulas were used to calculate the ORF1ab and RdRp log_10_ IU per milliliter for all positive samples.

### Data analysis.

A contingency table was prepared to assess overall agreement between Plex and Direct-Plex with 95% CIs using a Westgard QC 2 × 2 contingency calculator (Westgard QC, Madison, WI, USA). ORF1ab and RdRp *C_q_* values were compared with a two-tailed paired *t* test (*P* < 0.05). Similarly, the quantitative differences in IU per milliliter were also calculated. All statistical analyses were performed in Excel (Microsoft, Redmond, WA, USA) and MedCalc v15.4 (New York City, NY, USA).

### Institutional review board statement.

The residual samples used in the study were deidentified and results were not used to clinically manage patients (National Statement on Ethical Conduct in Human Research 2007 [May 2015], National Health and Medical Research Council, Australian Research Council, and Australian Vice-Chancellors’ Committee).
